# Influence of liberal versus conservative oxygen therapies on the hemodynamic parameters of mechanically ventilated patients with sepsis: a randomized clinical trial

**DOI:** 10.1186/s12871-024-02838-6

**Published:** 2024-12-20

**Authors:** Huda F. Ghazaly, Ahmed Alsaied A. Aly, Ahmed S. Tammam, Mahmoud M. Hassan, Soudy S. Hammad, Naggeh M. Mahmoud, Tarek S. Hemaida

**Affiliations:** 1 Anesthesia and Surgical Intensive Care Department, Faculty of Medicine, Aswan University, Aswan, Egypt; 2 Cardiology Department, Faculty of Medicine, Aswan University, Aswan, Egypt

**Keywords:** Cardiac output, Oxygen toxicity, Oxygen therapy, Sepsis, Stroke volume

## Abstract

**Background:**

There is no significant evidence verifying the efficacy of liberal versus conservative oxygen therapy on hemodynamics in patients with sepsis. We investigated how liberal and conservative oxygen therapy influenced stroke volume, cardiac output, and vasopressor needs in patients with sepsis undergoing mechanical ventilation.

**Methods:**

This randomized clinical trial included 106 patients with an admission diagnosis of infection, a Sequential Organ Failure Assessment (SOFA) score of two points or higher and required invasive mechanical ventilation for at least 72 h. Patients were randomly assigned to one of two oxygenation strategies: liberal (*n* = 53) with a target SpO2 of ≥ 96% or conservative (*n* = 53) with a target SpO2 of 88–92%. Transthoracic Doppler echocardiography was done twice to measure stroke volume and cardiac output, initially upon enrollment in the trial and then 72 h later. The primary outcome was stroke volume. Secondary outcomes were cardiac output, vasopressor use, mechanical ventilation duration, ICU stay length, and adverse events.

**Results:**

Stroke volume and cardiac output measurements did not differ significantly between research groups after 72 h of oxygenation treatment (*p* = 0.459 and 0.637, respectively). Forty-five patients (84.9%) in the conservative oxygen therapy group needed vasopressors to maintain their mean arterial pressure above 65 mmHg, whereas 35 patients (66.0%) in the liberal group did (*p* = 0.024). A multivariate logistic regression analysis of the independent variables for vasopressor requirements revealed that patients in the conservative oxygen group were 3.83 times more likely to require vasopressors (AOR = 3.83, 95% CI: 1.31–11.18, *p* = 0.014) than those in the liberal group. Older patients (AOR = 1.03, 95% CI: 1.01–1.07, *p* = 0.038) and those with higher SOFA scores (AOR = 1.36, CI: 1.09–1.68, *P* = 0.005) were significantly more likely to need vasopressors.

**Conclusions:**

Liberal or conservative oxygen therapy did not influence stroke volume or cardiac output measurements in mechanically ventilated patients with sepsis. Patients in the conservative oxygen group were more likely to require vasopressors than those in the liberal group.

**Trial registration:**

This study was approved by the Ethics Committee of Aswan University Hospital (approval number: Aswu/460/5/20) (registration date: 05/05/2020) and registered on ClinicalTrials.gov (NCT04824703) (03/30/2021).

## Introduction

Sepsis and septic shock remain significant global health burdens, characterized by elevated morbidity and mortality rates [1]. Sepsis is an unregulated host response to infection that results in tissue hypoperfusion and end-organ damage [2]. Optimizing oxygen delivery is crucial for patients with sepsis, particularly those on mechanical ventilation, which requires increasing cardiac output and oxygen supplementation [3]. Bak et al. [4] identified a negative correlation between increasing arterial oxygen levels and decreasing left ventricular stroke volume and end-diastolic area. The study involved nine healthy volunteers between the ages of 23 and 48. Previous research has also shown that oxygen therapy can considerably impact cardiovascular function [5]. Additionally, hyperoxia and oxidative imbalance produce excess reactive oxygen species (ROS), contributing to organ damage and cardiac cell death. Sepsis also enhances the formation of these free radicals [6]. Thus, the optimal oxygen regimen for patients with sepsis is still being determined.

A recent post-hoc analysis of data from 251 mechanically ventilated patients with sepsis who participated in a trial evaluating conservative to standard oxygen therapy revealed that conservative oxygen therapy did not result in a statistically significant reduction in 90-day mortality compared to standard oxygen therapy. However, the study was insufficiently powered to detect the hemodynamically meaningful advantage or harm of conservative oxygen therapy in this patient population [7]. Therefore, there is a lack of evidence verifying the effect of liberal versus conservative oxygen therapy on hemodynamics in mechanically ventilated patients with sepsis [8]. Notably, echocardiographic monitoring has recently been implemented in critical care, and stroke volume (SV) is becoming a more significant hemodynamic parameter to evaluate heart pump performance and organ perfusion. SV is considered the fundamental determinant of cardiac output (CO) and is less affected by compensatory processes [9]. 

This randomized clinical trial compared the effect of liberal versus conservative oxygen therapy on stroke volume and cardiac output in sepsis patients undergoing mechanical ventilation. Vasopressor needs, duration of mechanical ventilation, ICU length of stay, and ICU mortality were also evaluated.

## Patients and methods

### Ethics and registration

This study was approved by the Ethics Committee of Aswan University Hospital (approval number: Aswu/460/5/20) (registration date: 05/05/2020) and registered on ClinicalTrials.gov (NCT04824703) (03/30/2021). It was carried out in the surgical intensive care unit (SICU) at Aswan University Hospital and adhered to the Consolidated Standards of Reporting Trials guidelines. Patients or surrogates signed a written informed consent after learning about the trial. All methods were carried out according to the ethical standards outlined in the 1964 Declaration of Helsinki and subsequent amendments.

### Patient inclusion and exclusion criteria

This study included patients ≥ 18 years old admitted to the SICU with infection, SOFA score of two points or higher (indicating sepsis according to the Sepsis-3 diagnosis) [10], and expected to be mechanically ventilated for at least 72 h. Patients under the age of 18, pregnant women, those with acute respiratory distress syndrome, chronic obstructive pulmonary disease, idiopathic pulmonary fibrosis, cardiovascular disease, chronic kidney disease, and those who declined to participate were excluded from the study.

### Randomization

Patients were randomly assigned to one of two equal groups using computer-generated randomization tables. The group allocation was concealed in serially numbered, sealed, and opaque envelopes, which were opened at allocation time. The liberal oxygen therapy group (*n* = 53) included patients with target arterial oxygen saturation measured by pulse oximetry (SpO2) ≥ 96% and target arterial partial pressure of oxygen (PaO2) between 90 and 105 mm Hg, with no specific measures limiting the fraction of inspired oxygen (FiO2). FiO2 values less than 0.3 were not used.

The conservative oxygen therapy group (*n* = 53) included patients with target arterial oxygen saturation measured by pulse oximetry (SpO2) between 88 and 92% and target PaO2 between 60 and 75 mm Hg. The FiO2 was reduced as much as possible to a minimum of 0.21 while maintaining the SpO2 above the acceptable lower limit. If SpO2 ˃ 92%, FiO2 was decreased by 0.1 at intervals of no more than 5 min until SpO2 < 92%. If SpO2 was within target, FiO2 was reduced by 0.05 at 30-minute intervals until it reached 0.21 or SpO2 approached 88%. If SpO2 < 88%, immediately revert to the previous FiO2 to meet the target SpO2. If arterial blood gas shows PaO2 < 60 mmHg, FiO2 was increased regardless of SpO2 level. Patients received their assigned oxygen strategy until discharge from the ICU. We used PaO2 and SpO2 readings to influence FiO2 levels during the trial [11]. 

All patients were mechanically ventilated using an assist control pressure control (A/C PC) mode to deliver a tidal volume of 6–8 mL/kg, a respiratory rate of 12–16 cycles/min, an inspiratory to expiratory ratio of 1:2, and a PEEP of 5 cmH2O using (CARESCAPE R860, Model G1500197, Datex Ohmeda, Madison, USA). To maintain a Richmond Agitation Sedation Scale (RASS) score of -2 to -4 during the first 72 h of mechanical ventilation, patients were given dexmedetomidine infusions of 0.2 to 0.7 µg/kg/h and fentanyl infusions of 1.0 to 2.0 µg/kg/h, as per the institutional sedation/analgesia protocol. The study group allocation was unknown to the patients, their families, the transthoracic echocardiography operator, and those analyzing the results.

### The following clinical data were gained upon admission

- History and baseline characteristic data (age, gender, source of ICU admission, and sites of infection), complete laboratory investigations, microbiological samples, and radiographic investigations. Antimicrobials were given immediately within one hour of sepsis identification following the international recommendations [12].
- Sequential Organ Failure Assessment (SOFA) [13], Simplified Acute Physiology Score Ш (SAPS Ш) [14, 15], and Acute Physiology, Age, and Chronic Health Evaluation Π (APACHE Π) [16] scores were calculated upon ICU admission.
- Continuous arterial blood pressure monitoring was performed using an arterial catheter, and the heart rate was also continually measured using electrocardiography.
- After adequate fluid resuscitation, if diastolic arterial pressure (DAP) was lower than 60 mmHg or mean arterial blood pressure (MAP) was lower than 65 mmHg, we immediately initiated vasopressor (norepinephrine) infusion. The norepinephrine dosage was adjusted between 0.2 and 1.5 µg/kg/minute to maintain MAP above 65 mmHg and DBP above 60 mmHg [19].
- The central venous oxygen saturation (ScvO2) was measured every 12 h to guide fluid resuscitation. ScvO2 levels were characterized as high if they exceeded 75%, normal if they ranged between 65 and 75%, and low if they were less than 65% [17, 18].
- Arterial blood gas was checked every six hours. Mean FiO2, SpO2, PaO2, and the PaO2/FiO2 ratio were calculated daily.

### Stroke volume and cardiac output measurements

A single certified cardiologist, blind to group assignment, performed transthoracic Doppler echocardiography, which was repeated 72 h later to verify the impact of liberal versus conservative oxygen therapies on stroke volume and cardiac output. To improve the quality and consistency of echocardiographic interpretations and minimize intra-observer variability, measurements were repeated by the same operator to ensure real data. Image acquisition was performed following a standardized protocol based on the American Society of Echocardiography guidelines [20]. 

Standard echocardiographic views were acquired using the GE M5Sc Matrix Phased Array 1–5 MHz Ultrasound Probe (Vivid E95, GE Vingmed Ultrasound AS, Horten, Norway). Pulsed-wave Doppler samples were obtained in the center of the left ventricular outflow tract (LVOT) using the apical 5-chamber view to measure the velocity of blood passing through it. The angle between the Doppler signal and the aortic blood flow was kept as close to 0° as possible. This was important since the quality of the Doppler ultrasonography significantly depended on the alignment with the aortic blood flow and catching the signal at the correct angle. The velocity time integral (VTI) was determined by tracing the outline of the LVOT velocity waveform. The average VTI was calculated by tracing five consecutive Doppler velocity curves in five cardiac cycles. Using the long parasternal view, the LVOT diameter was determined by measuring the distance between the bases of the aortic valve cusp during systole. Three measurements were taken to determine the average LVOT diameter. LVOT area = [(LVOT diameter average / 2)^2^] × 3.14 [21. Stroke volume was calculated by multiplying the LVOT area by the VTI of the LVOT blood flow. Cardiac output was computed by multiplying the SV by the heart rate recorded during the measurement [22, 23]. 

### Outcome measures

The primary outcome was the stroke volume after 72 h of oxygen therapy. Secondary outcomes were cardiac output, the use of vasopressors, duration of mechanical ventilation, ICU length of stay, and ICU mortality.

### Sample size and statistical analysis

The sample size was estimated using G*Power software version 3.1.3. The primary outcome was the stroke volume after 72 h of oxygen therapy. Using a t-test for comparison and hypothesizing an effect size of 0.5 (difference in stroke volume between the two study groups), an alpha error probability of 0.05, a power (1-beta error probability) of 0.80, and an allocation ratio of 1:1, the minimal sample size was 102 patients (51 in each group).

Data was analyzed using the Statistical Package for Social Science (SPSS), version 26.0 for Windows. Qualitative data were presented as frequency and percentage. Quantitative data were presented as mean ± SD or median and interquartile range according to the normality of the data after testing its distribution by the Shapiro-Wilk test. The missing data was not imputed.

The Chi-square/Fisher Exact test was used to compare the proportions between the conservative and liberal oxygen therapy groups in the septic mechanically ventilated patients. Independent Sample T-test/Mann Whitney U test was used to compare the mean/median differences between groups. One-way repeated measures ANOVA compares the mean difference within each study group over time. Two-way repeated measures ANOVA compares the effect of time between groups. Pearson correlation was utilized to explore the correlation between stroke volume after 72 h of oxygen therapy and other variables. A univariate logistic regression analysis was employed to identify possible factors associated with mortality and vasopressor use in septic mechanically ventilated patients. The significant variables were then entered into a multivariate logistic regression analysis to calculate the adjusted odds ratio (AOR). P-values less than 0.05 were considered significant.

## Results

**Fig. 1:**
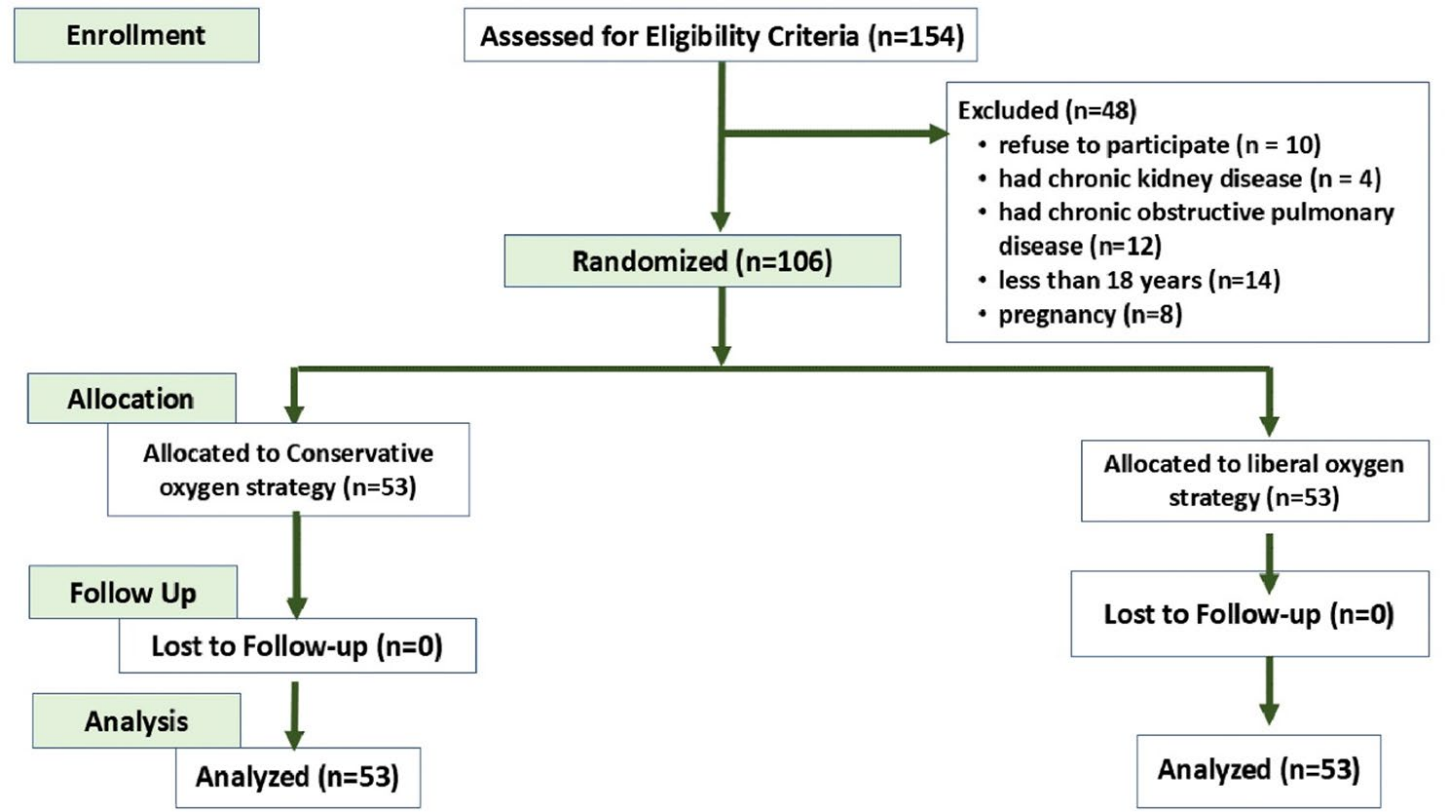
Study flow diagram

The study included 154 patients admitted to the SICU with infection, a SOFA score of two or more, and likely to be mechanically ventilated for at least 72 h. Forty-eight participants were excluded from the trial due to refusal to participate (*n* = 10), chronic kidney disease (*n* = 4), chronic obstructive pulmonary disease (*n* = 12), age under 18 (*n* = 14), or pregnancy (*n* = 8). Finally, 106 patients participated in the trial. They were randomly assigned to either the conservative oxygen strategy group (*n* = 53) or the liberal oxygen strategy group (*n* = 53) (Fig. 1). Patient characteristics and admission severity sources were comparable between the study groups (Table 1).

**Table 1:** Characteristic data of study groups

Variables	Conservative oxygen strategy (*n* = 53)	Liberal oxygen strategy (*n* = 53)	*P*-value
Age (years)	48.04 ± 12.25	52.60 ± 16.21	0.105
Gender, male	36 (67.9%)	33 (62.3%)	0.541
Source of admission to ICU			
• Emergency room	29 (54.7%)	22 (41.5%)	0.261
• Operation room	15 (28.3%)	20 (37.7%)	
• Ward	7 (13.2%)	5 (9.4%)	
• Referred from other hospital	2 (3.8%)	6 (11.3%)	
Sites of infection			
Abdomen			
• Gastrointestinal perforation	9 (17.0%)	9 (17.0%)	
• Acute pancreatitis	10 (18.9%)	7 (13.2%)	
• Biliary tract infection	7 (13.2%)	8 (15.1%)	
Thorax			
• Pneumonia	11 (20.8%)	13 (24.5%)	0.967
Soft tissues			
• Surgical site/wound infection	13 (24.5%)	12 (22.6%)	
• Necrotizing fasciitis	3 (5.7%)	4 (7.5%)	
SAPS Ш score			
• Mean ± SD.	124.85 ± 57.79	111.58 ± 66.32	0.255
• Median (range)	136.0 (21–217)	106.0 (19–216)	
SOFA score			
• Mean ± SD.	7.47 ± 2.92	7.49 ± 3.57	0.659
• Median (range)	7.0 (3–15)	7.0 (3–15)	
APACHE Π score			
• Mean ± SD.	18.75 ± 9.28	16.42 ± 6.76	0.359
• Median (range)	16.0 (7–40)	15.0 (7–38)	

Data expressed as mean ± SD, median (range), or frequency (%). ICU, intensive care unit; SAPS Ш, Simplified Acute Physiology Score; SOFA, Sequential Organ Failure Assessment; APACHE Π, Acute Physiology and Chronic Health Evaluation

There were no significant differences in MBP (69.09 ± 8.68 vs. 71.37 ± 8.70; *p* = 0.179) or HR (103.49 ± 22.86 vs. 104.13 ± 23.38; *p* = 0.887) between conservative and liberal oxygen treatments (Table 2). Stroke volumes (57.28 ± 10.60 vs. 56.28 ± 10.06; *p* = 0.619) and cardiac output values (5.77 ± 1.03 vs. 5.71 ± 1.03; *p* = 0.772) did not differ significantly between conservative and liberal groups after 72 h of oxygen treatment. However, in the conservative oxygen group, SV showed a negative association with FiO2 (*p* = 0.014, *r* = -0.336) and a positive correlation with the PaO2/FiO2 ratio (*p* = 0.010, *r* = 0.350), implying that SV increased when FiO2 decreased or the PaO2/FiO2 ratio increased.

**Table 2:** Primary and secondary outcomes

Parameters	Conservative oxygen therapy (*n* = 53)	Liberal oxygen therapy (*n* = 53)	*P*-value
Mean blood pressure (mmHg)	69.09 ± 8.68	71.37 ± 8.70	0.179
Heart rate (bpm)	103.49 ± 22.86	104.13 ± 23.38	0.887
Stroke volume (ml)			
Upon enrollment in the trial	57.28 ± 10.60	56.28 ± 10.06	0.619
72 h after oxygen therapy	58.26 ± 10.62	56.79 ± 9.72	0.459
Cardiac output (L/min)			
Upon enrollment in the trial	5.77 ± 1.03	5.71 ± 1.03	0.772
72 h after oxygen therapy	5.85 ± 1.00	5.75 ± 1.04	0.637
Vasopressors needs	45 (84.9%)	35 (66.0%)	0.024
ICU length of stay (days)			
• Mean ± SD	14.00 ± 5.70	13.38 ± 5.47	0.498
• Median (range)	13.0 (3–25)	12.0 (4–25)	
Duration of mechanical ventilation (days)			
• Mean ± SD	9.85 ± 3.68	9.04 ± 3.80	0.260
• Median (range)	10.0 (3–15)	10.0 (3–15)	
Adverse events			
• ICU mortality			
Survived	36 (67.9%)	39 (73.6%)	0.522
Non-Survived	17 (32.1%)	14 (26.4%)	
• VAP	28 (52.8%)	19 (35.8%)	0.078
• Myocardial infarction	1 (1.9%)	2 (3.8%)	0.999
• RRT	3 (5.7%)	4 (7.5%)	0.999

Data expressed as mean ± SD, median (range), or frequency (%). ICU, intensive care unit; VAP, ventilator-associated pneumonia; RRT, renal replacement therapy

Vasopressors were needed by 45 patients (84.9%) in the conservative treatment group to keep their MAP above 65 mmHg, compared to 35 patients (66.0%) in the liberal treatment group (*p* = 0.024) (Table 2). The univariate logistic regression study of the independent variables for vasopressor needs in mechanically ventilated patients with sepsis revealed significant associations with increasing SOFA score, APACHE Π score, and the conservative oxygen management group. After adjusting for all variables, the multivariate logistic regression analysis revealed that the conservative oxygen group was 3.83 times more likely than the liberal group (reference) to require vasopressors (AOR = 3.83, 95% confidence interval [CI]: 1.31–11.18, *p* = 0.014). Elderly patients were significantly more likely to need vasopressors, with an AOR of 1.03 (95% CI: 1.01–1.07, *p* = 0.038). Furthermore, individuals with higher SOFA levels were significantly more likely to utilize vasopressors than those with lower values (AOR = 1.36, 95% CI, 1.09–1.68, *P* = 0.005) (Table 3).

**Table 3:** The logistic regression analysis of the independent variables for vasopressor requirements in sepsis patients undergoing mechanical ventilation

The independent predictors	Univariate		Multivariate	
	OR (95% CI)	*P*-Value	AOR (95% CI)	*P*-Value
Age	1.02 (0.99–1.05)	0.185	1.03 (1.01–1.07)	0.038
Female gender	1.22 (0.49–3.06)	0.662		
Duration of mechanical ventilation	1.00 (0.89–1.13)	0.879		
Length of ICU stay	0.94 (0.87–1.02)	0.168		
SAPSШ score	1.00 (0.99–1.01)	0.635		
SOFA score	1.37 (1.12–1.68)	0.002	1.36 (1.09–1.68)	0.005
APACHE Π score	1.07 (1.01–1.15)	0.038		
Treatment groups				
Conservative oxygen therapy	2.89 (1.12–7.42)	0.027	3.83 (1.31–11.18)	0.014
Liberal oxygen therapy	Reference group			

OR, Odds Ratio; AOR, adjusted odds ratio; 95% CI, 95% confidence interval. SAPS Ш, Simplified Acute Physiology Score; SOFA, Sequential Organ Failure Assessment; APACHE Π, Acute Physiology and Chronic Health Evaluation

**Fig. 2:**
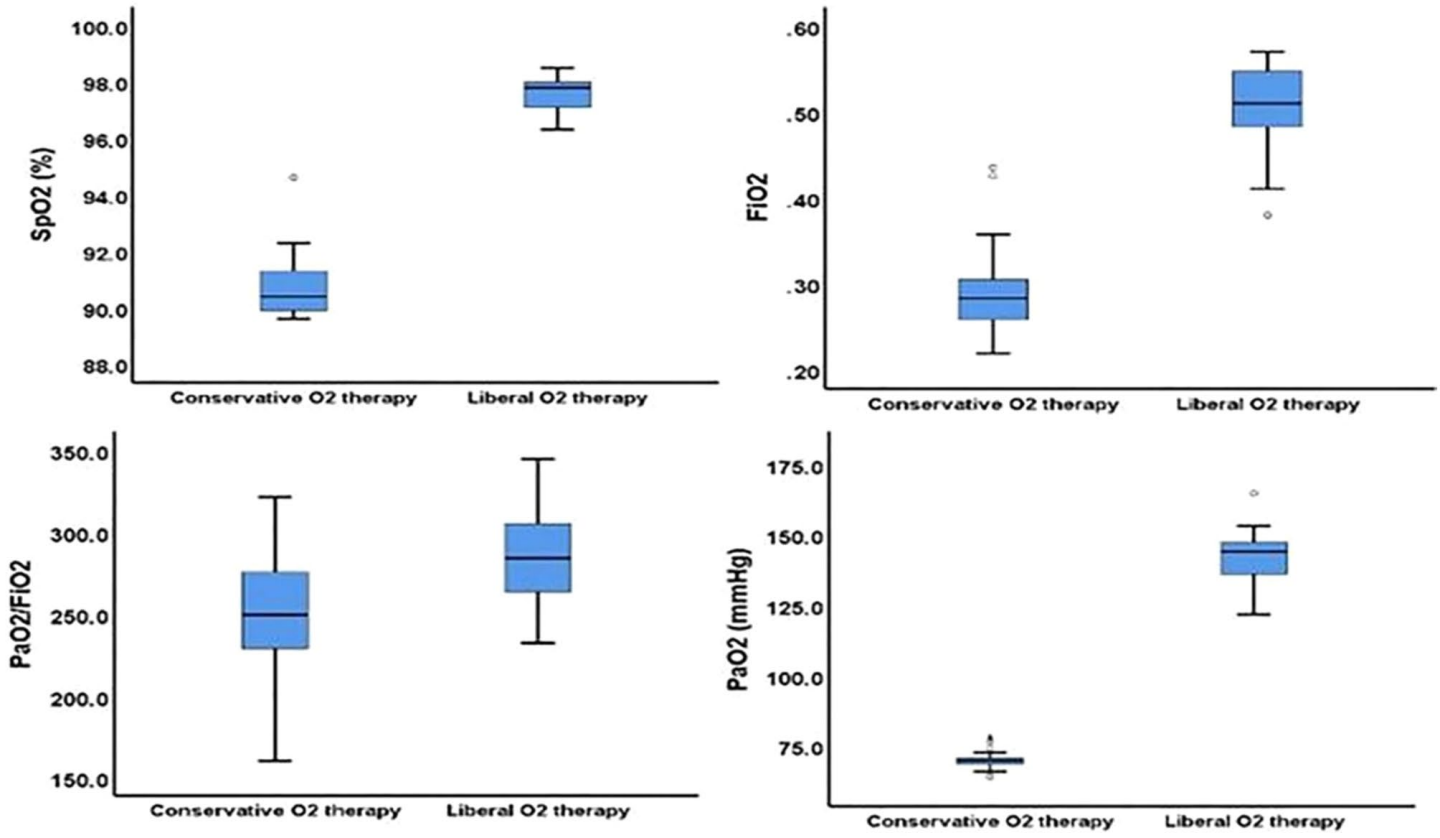
A Boxplot chart displays the oxygenation characteristics of liberal and conservative groups (the bar represents the median value and the 25th to 75th percentile)

**Fig. 3:**
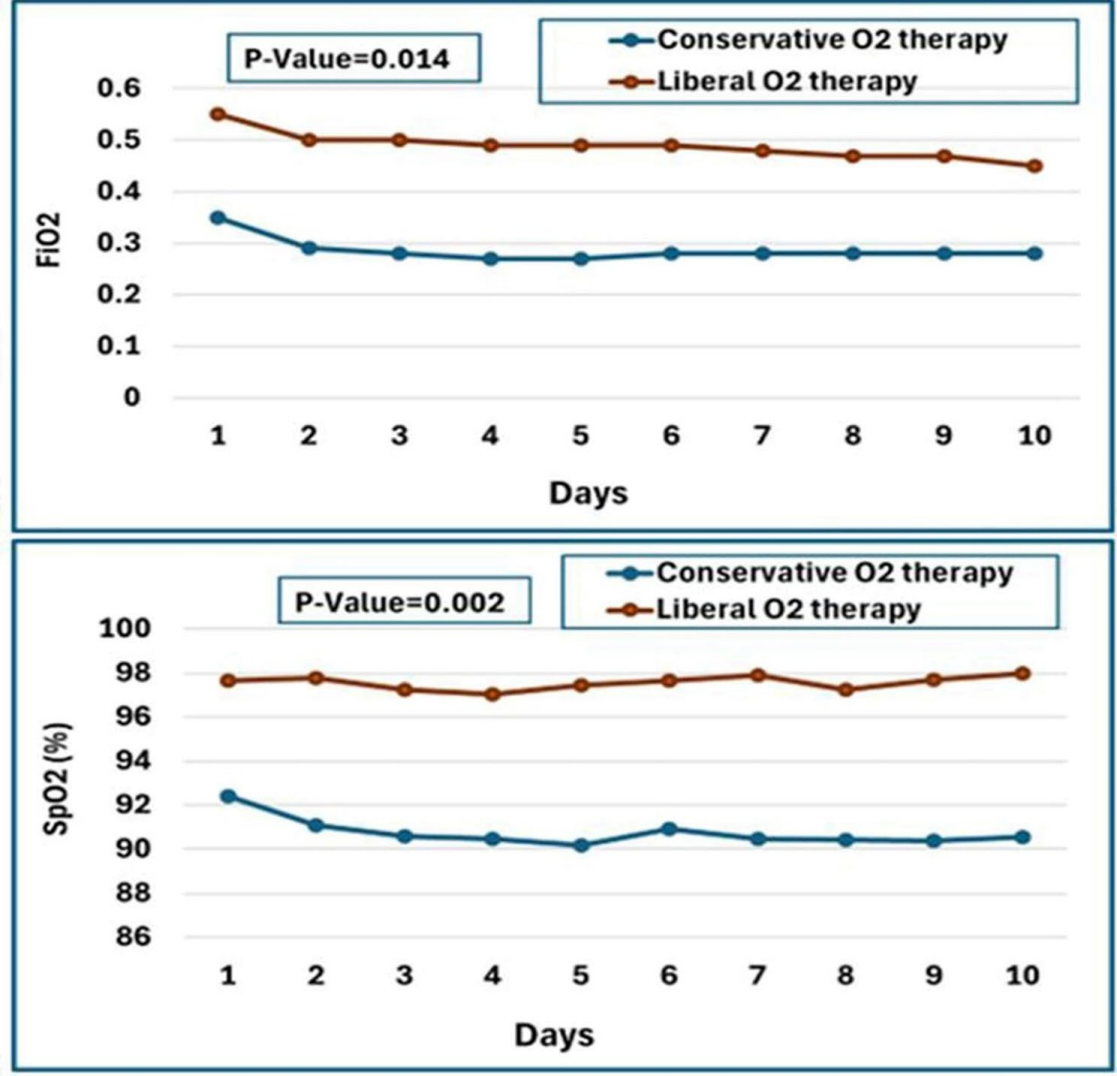
FiO2 and SpO2 levels in the conservative and liberal groups over the first ten days of mechanical ventilation

The median FiO2 used in the liberal versus conservative oxygenation groups was 0.51 (interquartile range [IQR], 0.48–0.55) vs. 0.28 (IQR, 0.25–0.30), SpO2 was 97.80% (IQR, 97.10–98.00%) vs. 90.40% (IQR, 89.90–91.30%), PaO2 was 144.20 mmHg (IQR, 132.90–148.00 mmHg) vs. 69.80 mmHg (IQR, 68.37–70.65 mmHg), and PaO2/FiO2 was 284.20 (IQR, 262.90–305.40) vs. 249.55 (IQR, 228.02–276.02) (Fig. 2). FiO2, SpO2, and PaO2 levels were higher in the liberal group compared to the conservative group over the first ten days of mechanical ventilation (*p* < 0.001). The mean FiO2 in liberal and conservative groups declined significantly (*p* = 0.001) from day one to day ten. Mean FiO2 levels significantly varied over time (*p* = 0.014) between the study groups. SpO2 levels decreased significantly (*p* < 0.001) in the conservative group between days one and ten, but not in the liberal group (*p* = 0.880). Mean SpO2 readings among research groups changed significantly over time (*p* = 0.002). The FiO2 and SpO2 values during the first ten days of mechanical ventilation are shown in Fig. 3.

**Fig. 4:**
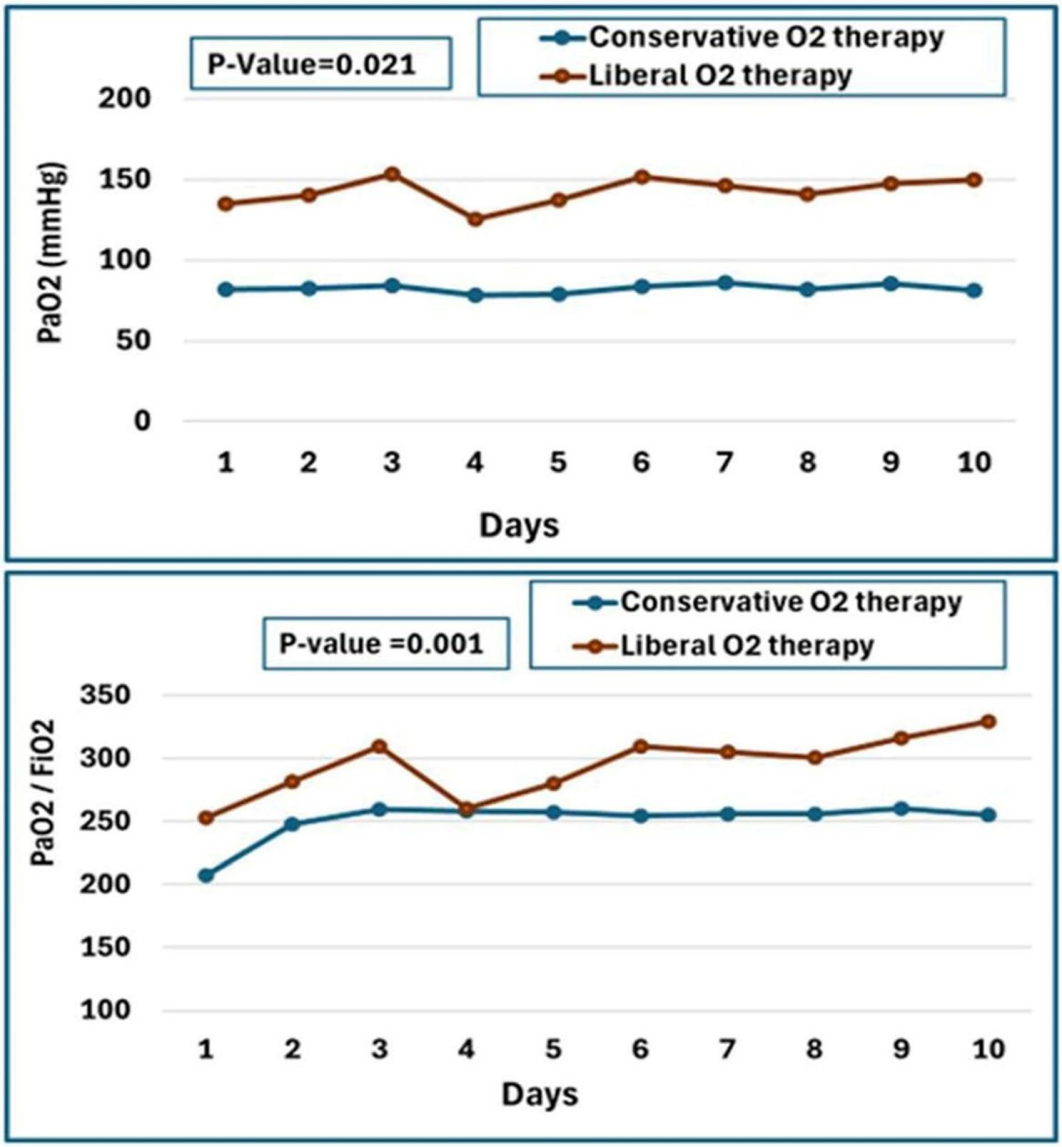
PaO2 and PaO2 / FiO2 ratios in the conservative and liberal groups throughout the first ten days of mechanical ventilation

The mean PaO_2_ levels increased significantly (*p* = 0.004) in the liberal group from day one to day ten, but not in the conservative group (*p* = 0.140). PaO2 levels differed significantly (*p* = 0.021) between study groups over time. The PaO2/FiO2 ratio increased (*p* = 0.001) in liberal and conservative groups between days one and ten. On day ten, the liberal group had a higher (*p* = 0.033) mean PaO2/FiO2 ratio than the conservative group. The mean PaO2/FiO2 ratio differed significantly between study groups over time (*p* = 0.001). The PaO2 values and PaO2/FiO2 ratio over the first ten days of mechanical ventilation are displayed in Fig. 4.

**Fig. 5:**
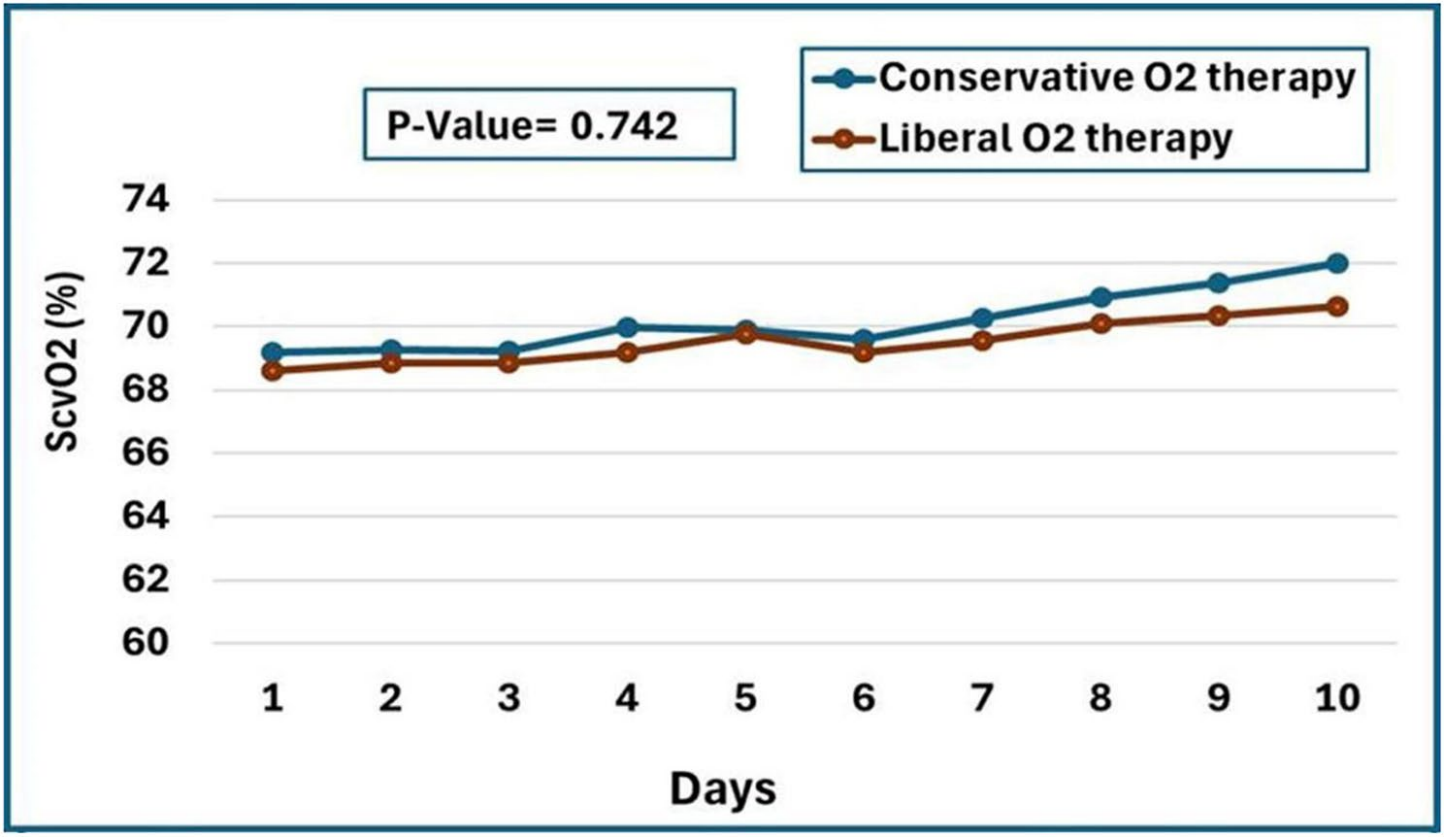
ScvO2 values in the conservative and liberal groups throughout the first ten days of mechanical ventilation

ScvO2 levels did not differ (*p* = 0.742) between the research groups during the first ten days of mechanical ventilation. The mean ScvO2 increased steadily in both conservative (*p* < 0.001) and liberal (*p* = 0.002) groups from day one to day ten (Fig. 5).

ICU mortality was similar among the study groups (*p* = 0.522). 17 patients (32.1%) in the conservative oxygen group and 14 patients (26.4%) in the liberal group died during their ICU stay (Table 2). Univariate logistic regression found that shorter ICU stays and higher SOFA and APACHE Π scores were significant determinants of ICU death. After adjusting for all variables, multivariate logistic regression analysis revealed that reduced ICU stays (AOR = 0.76: CI, 0.62–0.93, *P* = 0.008) and higher APACHE Π score (AOR = 1.60: CI, 1.28–2.00, *P* < 0.001) were significantly associated with death (Table 4).

**Table 4:** The logistic regression analysis of the independent predictors for mortality in sepsis patients undergoing mechanical ventilation

The independent predictors	Univariate		Multivariate	
	OR (95% CI)	*P*-Value	AOR (95% CI)	*P*-Value
Age	1.00 (0.97–1.03)	0.989		
Female gender	0.96 (0.42–2.32)	0.936		
Duration of mechanical ventilation	1.05 (0.93–1.17)	0.384		
Length of ICU stay	0.77 (0.69–0.87)	< 0.001	0.76 (0.62–0.93)	0.008
SAPSШ score	0.99 (0.99–1.01)	0.742		
SOFA score	4.89 (1.96–12.20)	0.001		
APACHE Π score	1.62 (1.30–2.01)	< 0.001	1.60 (1.28-2.00)	< 0.001
Treatment groups				
Conservative oxygen therapy	1.32 (0.56–3.04)	0.522		
Liberal oxygen therapy	Reference group			

OR, Odds Ratio; AOR, adjusted odds ratio; 95% CI, 95% confidence interval. SAPS Ш, Simplified Acute Physiology Score; SOFA, Sequential Organ Failure Assessment; APACHE Π, Acute Physiology and Chronic Health Evaluation

## Discussion

The current approach to oxygen administration in critical care is often liberal, which can serve as a buffer against hypoxia. However, the benefits of liberal oxygenation must be weighed against the hazards of tissue hyperoxia [24]. We conducted this trial to evaluate how different oxygen regimens influence hemodynamics in septic patients. We have found no significant differences in stroke volume or cardiac output measurements between the liberal and conservative regimens after 72 h of oxygen therapy. The conservative oxygen group had a larger proportion of patients who needed vasopressors than the liberal group. A multivariate logistic regression analysis of vasopressor needs revealed that the conservative oxygen group was 3.83 times more likely than the liberal group to require vasopressors. Additionally, older patients and those with higher SOFA scores were significantly more likely to use vasopressors. There were no significant differences between the liberal and conservative groups regarding mechanical ventilation duration, ICU stay, or ICU mortality. Higher APACHE Π scores and shorter ICU stays were significantly associated with mortality in a multivariate logistic regression analysis.

Excessive oxygen administration has a negative impact by producing free radicals, which generate an imbalance between oxidative and anti-oxidative species, alter cell metabolism, and inhibit enzyme activity [25]. Hyperoxia has also been associated with systemic vasoconstriction via reducing the synthesis and availability of nitric oxide, as well as a decrease in heart rate and a consequent reduction in cardiac output [26, 27]. Moreover, sepsis is strongly linked with impaired cardiovascular function [28]. It can cause vasodilatation, hypotension, and reduced global cardiac function [29]. Our clinical trial is the first to use stroke volume and cardiac output as monitoring parameters to compare the effect of liberal versus conservative oxygen therapies on hemodynamics in sepsis patients on mechanical ventilation.

Experts strongly recommend implementing critical care echocardiography as the first-line technique to assess the hemodynamic characteristics associated with shock, rather than invasive procedures [23]. Stroke volume and cardiac output indicate significant hemodynamic instability in ICU patients [30]. A previous transthoracic echocardiographic study included 106 patients admitted to three adult ICUs with sepsis or septic shock. The study found that 64% of patients with sepsis or septic shock have myocardial dysfunction, which includes left ventricular diastolic, systolic, and/or right ventricular failure [31]. Sepsis causes maximum cardiac depression 48 to 72 h after onset, which appears normal on the baseline echocardiogram [32]. In the current study, we evaluated SV upon enrollment in the trial and again 72 h later using the Doppler echocardiography approach, which correlates well with invasive SV and has high reliability [22]. We found no significant differences in heart rate, stroke volumes, or cardiac output values between the conservative and liberal treatment groups after 72 h of oxygen therapy [33]. An earlier study revealed that delivering 100% oxygen to individuals with congestive heart failure lowered cardiac output and stroke volume without affecting blood pressure or heart rate [34]. In line with our findings, Smit et al. found that hyperoxia did not significantly influence heart rate or cardiac output in sepsis patients. On the other hand, they reported that hyperoxia treatment reduced stroke volume and cardiac output in patients with heart failure by 8.6% and 15.2%, respectively. These discrepancies could be attributed to the various types of patients with different illnesses.

Hyperoxia enhances systemic vasoconstriction by producing significant levels of reactive oxygen species, which inhibit endothelial-derived nitric oxide (NO) and limit NO bioavailability [35, 36]. In contrast, a decrease in local O2 tension (PO2) in systemic arteries typically results in vasodilation [37, 38]. In our study, we observed that more patients in the conservative group (84.9%) needed vasopressors than those in the liberal group (66.0%). These findings support previous data indicating that hyperoxia-induced peripheral vasoconstriction may alleviate circulatory shock and decrease fluid and vasopressor resuscitation requirements [39–41]. However, a recent meta-analysis comprising three studies on septic patients found that hyperoxia did not affect systemic vascular resistance in this cohort [33]. Asfar et al. discovered no differences in vasopressor requirements when patients with septic shock were ventilated with 100% oxygen.^42^

A multicenter randomized clinical trial (HYPERS2S) enrolled patients with septic shock undergoing mechanical ventilation. They reported that setting FiO2 to 1.0 to produce arterial hyperoxia may increase the risk of 28-day mortality. Out of 434 patients, 93 (43%) died in the hyperoxia group, while 77 (35%) died in the normoxia group [42]. Chu and colleagues [43] conducted a meta-analysis of 25 randomized controlled trials, including patients with sepsis, critical illness, and emergency surgery. They revealed that liberal oxygen therapy (median FiO2 0.52) was hazardous, with an increased risk of both short- and long-term mortality compared to conservative therapy (median FiO2 0.21). The present research found no significant differences in ICU mortality between the study groups. These findings are comparable with a recent study on 2541 critically ill patients getting invasive mechanical ventilation. The study found that 281 patients (34.8%) in the lower-target group and 290 patients (33.2%) in the higher-target group died within 28 days [44]. In a study of 103 adult patients receiving invasive mechanical ventilation, Panwar et al. [40] found no mortality difference between conservative and liberal oxygenation strategies. They concluded that a conservative approach was more feasible than a liberal one. Young et al. [7] on the other hand, discovered that the conservative oxygen technique increased 90-day mortality in septic patients by 7% compared with liberal oxygen treatment, but the difference was not statistically significant. Differences in research design and oxygenation categorization may contribute to discrepancies in mortalities among ventilated ICU patients.

Our study groups had similar characteristics and sources of sepsis (*p* > 0.05). Current efforts are focused on creating subgroup patient categorization within the sepsis population to provide a personalized, specific treatment approach. These subgroups can be identified using clinical data, organ dysfunction patterns, vital signs, and laboratory results [45]. A recent study evaluated clinical sub-phenotypes, including heart rate, respiratory rate, and blood sodium levels, to identify patients at risk of developing postoperative sepsis after laparoscopic surgery for gastrointestinal perforations and provide specific treatment strategies [46]. 

The study has some limitations. First, we only included sepsis patients on mechanical ventilation in a single SICU, which may limit the generalizability of our findings. Second, considering the heterogeneity of the study population and their sources of sepsis, more work is needed to investigate how the various subgroups of patients can provide different results and conclusions. Third, laboratory findings were collected but not analyzed because the study lacked adequate power to identify statistically significant differences between laboratory results. Fourth, we did not collect data on antibiotic use or infection source management strategies. Fifth, we did not do serial measurements of stroke volume and cardiac outputs since many patients had been weaned off mechanical ventilation by 72 h.

In conclusion, both liberal and conservative oxygen treatment had no significant impact on stroke volume or cardiac output after 72 h of oxygenation therapy in sepsis patients undergoing mechanical ventilation. A greater number of patients in the conservative oxygen group required vasopressors than in the liberal group. The conservative oxygen treatment group was 3.83 times more likely to use vasopressors than the liberal group. Thus, liberal oxygenation might be preferable for this target population.

## Data Availability

All data used and analyzed in this study are available from the corresponding authors upon reasonable request.

## Abbreviations

ROS: Reactive oxygen species
SV: Stroke volume
CO: Cardiac output
SICU: Surgical intensive care unit
SpO2: Arterial oxygen saturation
PaO2: Arterial partial pressure of oxygen
FiO2: Fraction of inspired oxygen
ScvO2: Central venous oxygen saturation
SOFA: Sequential Organ Failure Assessment
SAPS: Simplified Acute Physiology Score
APACHE: Acute Physiology, Age, and Chronic Health Evaluation
LVOT: Left ventricular outflow tract
VTI: Velocity time integral
LVOT VTI: Left ventricular outflow tract velocity time integral

## References

[1] La Via L, Sangiorgio G, Stefani S, et al. The global burden of Sepsis and Septic Shock. Epidemiologia. 2024;5(3):456–78. 10.3390/epidemiologia5030032. 39189251 10.3390/epidemiologia5030032 PMC11348270

[2] Khalid N, Patel PD, Alghareeb R, Hussain A, Maheshwari MV. The Effect of Sepsis on myocardial function: a review of Pathophysiology, Diagnostic Criteria, and treatment. Cureus. 2022;14(6). 10.7759/cureus.26178. 10.7759/cureus.26178 PMC9306401 35891864

[3] Russell A, Rivers EP, Giri PC, Jaehne AK, Nguyen HB. A physiologic approach to hemodynamic monitoring and optimizing oxygen delivery in shock resuscitation. J Clin Med. 2020;9(7):1–18. 10.3390/jcm9072052. 10.3390/jcm9072052 PMC7408843 32629778

[4] Bak Z, Sjöberg F, Rousseau A, Steinvall I, Janerot-Sjoberg B. Human cardiovascular dose–response to supplemental oxygen. Acta Physiol. 2007;191(1):15–24. 10.1111/j.1748-1716.2007.01710.x 17506865

[5] Waring WS, Thomson AJ, Adwani SH, et al. Cardiovascular effects of acute oxygen administration in healthy adults. J Cardiovasc Pharmacol. 2003;42(2):245–50. 10.1097/00005344-200308000-00014. 12883329 10.1097/00005344-200308000-00014

[6] Soriano FG, Nogueira AC, Caldini EG, et al. Potential role of poly(adenosine 5′-diphosphate-ribose) polymerase activation in the pathogenesis of myocardial contractile dysfunction associated with human septic shock. Crit Care Med. 2006;34(4):1073–9. 10.1097/01.CCM.0000206470.47721.8D. 16484919 10.1097/01.CCM.0000206470.47721.8D

[7] Young P, Mackle D, Bellomo R, et al. Conservative oxygen therapy for mechanically ventilated adults with sepsis: a post hoc analysis of data from the intensive care unit randomized trial comparing two approaches to oxygen therapy (ICU-ROX). Intensive Care Med. 2020;46(1):17–26. 10.1007/s00134-019-05857-x. 31748836 10.1007/s00134-019-05857-x PMC7223684

[8] Barbateskovic M, Ol S. Oxygenation for adults admitted to the intensive care unit (review). Cochrane Database Syst Rev. 2019;1110.1002/14651858.CD012631.pub2.www.cochranelibrary.com. 10.1002/14651858.CD012631.pub3 PMC10498149 37700687

[9] Babbs CF. Noninvasive measurement of cardiac stroke volume using pulse wave velocity and aortic dimensions: a simulation study. Biomed Eng Online. 2014;13(1):1–25. 10.1186/1475-925X-13-137. 25238910 10.1186/1475-925X-13-137 PMC4271357

[10] Seymour CW, Liu VX, Iwashyna TJ, et al. Assessment of clinical criteria for sepsis for the third international consensus definitions for sepsis and septic shock (sepsis-3). JAMA - J Am Med Assoc. 2016;315(8):762–74. 10.1001/jama.2016.0288. 10.1001/jama.2016.0288 PMC5433435 26903335

[11] Yang X, Shang Y, Yuan S. Low versus high pulse oxygen saturation directed oxygen therapy in critically ill patients: a randomized controlled pilot study. J Thorac Dis. 2019;11(10):4234–40. 10.21037/jtd.2019.09.66. 31737308 10.21037/jtd.2019.09.66 PMC6837987

[12] Evans L, Rhodes A, Alhazzani W, et al. Surviving sepsis campaign: international guidelines for management of sepsis and septic shock 2021. Intensive Care Med. 2021;47(11):1181–247. 10.1007/s00134-021-06506-y. 34599691 10.1007/s00134-021-06506-y PMC8486643

[13] Vincent JL, Moreno R, Takala J, et al. The SOFA (Sepsis-related Organ failure Assessment) score to describe organ dysfunction/failure. Intensive Care Med. 1996;22(7):707–10. 10.1007/BF01709751. 8844239 10.1007/BF01709751

[14] Metnitz PGH, Moreno RP, Almeida E, et al. SAPS 3-From evaluation of the patient to evaluation of the intensive care unit. Part 1: objectives, methods and cohort description. Intensive Care Med. 2005;31(10):1336–44. 10.1007/s00134-005-2762-6. 16132893 10.1007/s00134-005-2762-6 PMC1315314

[15] Moreno RP, Metnitz PGH, Almeida E, et al. SAPS 3 - from evaluation of the patient to evaluation of the intensive care unit. Part 2: development of a prognostic model for hospital mortality at ICU admission. Intensive Care Med. 2005;31(10):1345–55. 10.1007/s00134-005-2763-5. 16132892 10.1007/s00134-005-2763-5 PMC1315315

[16] Knaus WA, Draper EA, Wagner DP, Zimmerman JE. APACHE II: a severity of disease classification system. Crit Care Med. 1985;13(10):818–29. 3928249

[17] Futier E, Robin E, Jabaudon M, et al. Central venous O2saturation and venous-to-arterial CO2difference as complementary tools for goal-directed therapy during high-risk surgery. Crit Care. 2010;14(5):1–11. 10.1186/cc9310. 10.1186/cc9310 PMC3219300 21034476

[18] Ford H, Systems H, Re-. CW. The Ne w E n g l a nd Jour n a l o f Me d ic i ne EARLY GOAL-DIRECTED THERAPY IN THE TREATMENT OF SEVERE SEPSIS AND SEPTIC SHOCK. 2001;345(19):1368–1377. 10.1056/NEJMoa010307 11794169

[19] Shi R, Hamzaoui O, Vita N, De, et al. Vasopressors in septic shock: which, when, and how much? Ann Transl Med. 2020;8(12):794–794. 10.21037/atm.2020.04.24. 32647719 10.21037/atm.2020.04.24 PMC7333107

[20] Mitchell C, Rahko PS, Blauwet LA, et al. Guidelines for performing a comprehensive transthoracic echocardiographic examination in adults: recommendations from the American Society of Echocardiography. J Am Soc Echocardiogr. 2019;32(1):1–64. 10.1016/j.echo.2018.06.004. 30282592 10.1016/j.echo.2018.06.004

[21] Mercado P, Maizel J, Beyls C, et al. Transthoracic echocardiography: an accurate and precise method for estimating cardiac output in the critically ill patient. Crit Care. 2017;21(1):1–8. 10.1186/s13054-017-1737-7. 28595621 10.1186/s13054-017-1737-7 PMC5465531

[22] Porter TR, Shillcutt SK, Adams MS, et al. Guidelines for the use of echocardiography as a monitor for therapeutic intervention in adults: a report from the American society of echocardiography. J Am Soc Echocardiogr. 2015;28(1):40–56. 10.1016/j.echo.2014.09.009. 25559474 10.1016/j.echo.2014.09.009

[23] Vignon P. Continuous cardiac output assessment or serial echocardiography during septic shock resuscitation? Ann Transl Med. 2020;8(12):797–797. 10.21037/atm.2020.04.11. 32647722 10.21037/atm.2020.04.11 PMC7333154

[24] Carvalho M, Soares M, Machado HS. Paradigms of Oxygen Therapy in critically ill patients. J Intensive Crit Care. 2017;03(01):1–6. 10.21767/2471-8505.100062.

[25] Patel DN, Goel A, Agarwal SB, Garg P, Lakhani KK. Oxygen toxicity. J Indian Acad Clin Med. 2003;4(3):234–7.

[26] Sjöberg F, Singer M. The medical use of oxygen: a time for critical reappraisal. J Intern Med. 2013;274(6):505–28. 10.1111/joim.12139. 24206183 10.1111/joim.12139

[27] Rousseau A, Bak Z, Janerot-Sjöberg B, Sjöberg F. Acute hyperoxaemia‐induced effects on regional blood flow, oxygen consumption and central circulation in man. Acta Physiol Scand. 2005;183(3):231–40. 15743383 10.1111/j.1365-201X.2005.01405.x

[28] Habimana R, Choi I, Cho HJ, Kim D, Lee K, Jeong I. Sepsis-induced cardiac dysfunction: a review of pathophysiology. Acute Crit Care. 2020;35(2):57–66. 10.4266/ACC.2020.00248. 32506871 10.4266/acc.2020.00248 PMC7280799

[29] Vieillard-Baron A, Caille V, Charron C, Belliard G, Page B, Jardin F. Actual incidence of global left ventricular hypokinesia in adult septic shock. Crit Care Med. 2008;36(6):1701–6. 18496368 10.1097/CCM.0b013e318174db05

[30] Jang GY, Jeong YJ, Zhang T, et al. Noninvasive, simultaneous, and continuous measurements of stroke volume and tidal volume using EIT: feasibility study of animal experiments. Sci Rep. 2020;10(1):1–12. 10.1038/s41598-020-68139-3. 32647206 10.1038/s41598-020-68139-3 PMC7347894

[31] Pulido JN, Afessa B, Masaki M et al. Clinical spectrum, frequency, and significance of myocardial dysfunction in severe sepsis and septic shock. In: *Mayo Clinic Proceedings*. Vol 87. Elsevier; 2012:620–628. 10.1016/j.mayocp.2012.01.018 PMC3538477 22683055

[32] Hunter JD, Doddi M. Sepsis and the heart. Br J Anaesth. 2010;104(1):3–11. 10.1093/bja/aep339. 19939836 10.1093/bja/aep339

[33] Smit B, Smulders YM, van der Wouden JC, Oudemans-van Straaten HM, Spoelstra-de Man AME. Hemodynamic effects of acute hyperoxia: systematic review and meta-analysis. Crit Care. 2018;22(1):1–10. 10.1186/s13054-018-1968-2. 29477145 10.1186/s13054-018-1968-2 PMC6389225

[34] Haque WA, Boehmer J, Clemson BS, Leuenberger UA, Silber DH, Sinoway LI. Hemodynamic effects of supplemental oxygen administration in congestive heart failure. J Am Coll Cardiol. 1996;27(2):353–7. 10.1016/0735-1097(95)00474-2. 8557905 10.1016/0735-1097(95)00474-2

[35] Waring WS, Thomson AJ, Adwani SH, et al. Cardiovascular Effects of Acute Oxygen Administration in healthy adults. J Cardiovasc Pharmacol. 2003;42(2):245–50. 12883329 10.1097/00005344-200308000-00014

[36] Hafner S, Beloncle F, Koch A, Radermacher P, Asfar P. Hyperoxia in intensive care, emergency, and peri-operative medicine: Dr. Jekyll or Mr. Hyde? A 2015 update. Ann Intensive Care. 2015;5(1):1–14. 10.1186/s13613-015-0084-6. 26585328 10.1186/s13613-015-0084-6 PMC4653126

[37] Sparks HV Jr. Effect of local metabolic factors on vascular smooth muscle. Compr Physiol Published Online 2011:475–513.

[38] Franco-Obregón A, López-Barneo J. Differential oxygen sensitivity of calcium channels in rabbit smooth muscle cells of conduit and resistance pulmonary arteries. J Physiol. 1996;491(2):511–8. 10.1113/jphysiol.1996.sp021235. 8866874 10.1113/jphysiol.1996.sp021235 PMC1158745

[39] Calzia E, Asfar P, Hauser B, et al. Hyperoxia may be beneficial. Crit Care Med. 2010;38(10):S559–68. 21164398 10.1097/CCM.0b013e3181f1fe70

[40] Panwar R, Hardie M, Bellomo R, et al. Conservative versus liberal oxygenation targets for mechanically ventilated patients: a pilot multicenter randomized controlled trial. Am J Respir Crit Care Med. 2016;193(1):43–51. 10.1164/rccm.201505-1019OC. 26334785 10.1164/rccm.201505-1019OC

[41] van der Wal LI, Grim CCA, van Westerloo DJ, Schultz MJ, de Jonge E, Helmerhorst HJF. Higher versus lower oxygenation strategies in the general intensive care unit population: a systematic review, meta-analysis and meta-regression of randomized controlled trials. J Crit Care. 2022;72(2022):154151. 10.1016/j.jcrc.2022.154151. 36182731 10.1016/j.jcrc.2022.154151

[42] Asfar P, Schortgen F, Boisramé-Helms J, et al. Hyperoxia and hypertonic saline in patients with septic shock (HYPERS2S): a two-by-two factorial, multicentre, randomised, clinical trial. Lancet Respir Med. 2017;5(3):180–90. 28219612 10.1016/S2213-2600(17)30046-2

[43] Chu DK, Kim LHY, Young PJ, et al. Mortality and morbidity in acutely ill adults treated with liberal versus conservative oxygen therapy (IOTA): a systematic review and meta-analysis. Lancet. 2018;391(10131):1693–705. 10.1016/S0140-6736(18)30479-3. 29726345 10.1016/S0140-6736(18)30479-3

[44] Semler MW, Casey JD, Lloyd BD, et al. Oxygen-saturation targets for critically ill adults receiving mechanical ventilation. N Engl J Med. 2022;387(19):1759–69. 10.1056/nejmoa2208415. 36278971 10.1056/NEJMoa2208415 PMC9724830

[45] Seymour CW, Kennedy JN, Wang S, et al. Derivation, validation, and potential treatment implications of Novel Clinical Phenotypes for Sepsis. JAMA - J Am Med Assoc. 2019;321(20):2003–17. 10.1001/jama.2019.5791. 10.1001/jama.2019.5791 PMC6537818 31104070

[46] Yang J, Zhang B, Hu C, et al. Identification of clinical subphenotypes of sepsis after laparoscopic surgery. Laparosc Endosc Robot Surg. 2024;7(1):16–26. 10.1016/j.lers.2024.02.001.

